# Discovering proteins for chemoprevention and chemotherapy by curcumin in liver fluke infection-induced bile duct cancer

**DOI:** 10.1371/journal.pone.0207405

**Published:** 2018-11-15

**Authors:** Jarinya Khoontawad, Kitti Intuyod, Rucksak Rucksaken, Nuttanan Hongsrichan, Chawalit Pairojkul, Porntip Pinlaor, Thidarut Boonmars, Chaisiri Wongkham, Alun Jones, Jordan Plieskatt, Jeremy Potriquet, Jeffrey M. Bethony, Jason Mulvenna, Somchai Pinlaor

**Affiliations:** 1 Department of Parasitology, Faculty of Medicine, Khon Kaen University, Khon Kaen, Thailand; 2 Cholangiocarcinoma Research Institute, Faculty of Medicine, Khon Kaen University, Khon Kaen, Thailand; 3 Department of Thai Traditional Medicine, Faculty of Natural Resources, Rajamangala University of Technology Isan, Sakon Nakhon Campus, Sakon Nakhon, Thailand; 4 Department of Veterinary Technology, Faculty of Veterinary Technology, Kasetsart University, Bangkok, Thailand; 5 Department of Pathology, Faculty of Medicine, Khon Kaen University, Khon Kaen, Thailand; 6 Centre for Research and Development in Medical Diagnostic Laboratory, Faculty of Associated Medical Sciences, Khon Kaen University, Khon Kaen, Thailand; 7 Department of Biochemistry, Faculty of Medicine, Khon Kaen University, Khon Kaen, Thailand; 8 The University of Queensland, Institute for Molecular Bioscience, Brisbane, Queensland, Australia; 9 Department of Microbiology, Immunology and Tropical Medicine, School of Medicine and Health Sciences, George Washington University, Washington, DC, United States of America; 10 Research Center for Neglected Diseases of Poverty, School of Medicine and Health Sciences, George Washington University, Washington, DC, United States of America; 11 Australian Institute of Tropical Health & Medicine, James Cook University, Cairns, Queensland, Australia; 12 The University of Queensland, School of Biomedical Sciences, Brisbane, Queensland, Australia; University of South Alabama Mitchell Cancer Institute, UNITED STATES

## Abstract

Modulation or prevention of protein changes during the cholangiocarcinoma (CCA) process induced by *Opisthorchis viverrini* (Ov) infection may become a key strategy for prevention and treatment of CCA. Monitoring of such changes could lead to discovery of protein targets for CCA treatment. Curcumin exerts anti-inflammatory and anti-CCA activities partly through its protein-modulatory ability. To support the potential use of curcumin and to discover novel target molecules for CCA treatment, we used a quantitative proteomic approach to investigate the effects of curcumin on protein changes in an Ov-induced CCA-harboring hamster model. Isobaric labelling and tandem mass spectrometry were used to compare the protein expression profiles of liver tissues from CCA hamsters with or without curcumin dietary supplementation. Among the dysregulated proteins, five were upregulated in liver tissues of CCA hamsters but markedly downregulated in the CCA hamsters supplemented with curcumin: S100A6, lumican, plastin-2, 14-3-3 zeta/delta and vimentin. Western blot and immunohistochemical analyses also showed similar expression patterns of these proteins in liver tissues of hamsters in the CCA and CCA + curcumin groups. Proteins such as clusterin and S100A10, involved in the NF-κB signaling pathway, an important signaling cascade involved in CCA genesis, were also upregulated in CCA hamsters and were then suppressed by curcumin treatment. Taken together, our results demonstrate the important changes in the proteome during the genesis of *O*. *viverrini*-induced CCA and provide an insight into the possible protein targets for prevention and treatment of this cancer.

## Introduction

Curcumin, a yellow pigment compound found in the plant *Curcuma longa* L., is a hydrophobic polyphenol which exhibits a variety of therapeutic properties but principally anti-inflammatory and anti-cancer effects [1, 2]. These effects have been clearly demonstrated in both *in vivo* and *in vitro* experiments using inflammation-linked cancer models as well as in clinical trials [3–5].

Cholangiocarcinoma (CCA), a malignant tumor originating from biliary epithelium cells, is a rare cancer in Western countries but is highly prevalent in Southeast Asian countries, especially in Northeast Thailand (>85 per 100,000 population) [6], where co-occurrence with small liver-fluke (*Opisthorchis viverrini*) infection has been observed [7]. Chronic inflammation caused by infection with *O*. *viverrini* is strongly associated with the tumorigenesis [8, 9]. Increased production of reactive oxygen (ROS) and nitrogen species (NOS) by host cells in response to infection are not only toxic to the parasites but also cause the modification of biomolecules such as DNA, proteins and lipids, ultimately leading to CCA tumorigenesis [9]. Chemotherapy using gemcitabine-based regimens represents the first line treatment of unresectable CCA patients since almost all of whom are diagnosed at a late stage and cannot be cured effectively by surgery [10, 11]. However, gemcitabine treatment results in multiple adverse events and the disease develops resistance to the drug over time [11]. Hence, using phytochemical substances with anti-inflammatory and anti-cancer properties could be very valuable for prevention and treatment of CCA. We have previously reported that dietary administration of curcumin reduces CCA incidence, retards tumor growth and prolongs the survival of animals in an *in vivo* model [12], and also exerts cytotoxicity against CCA cell lines *in vitro* [13].

Mechanistically, curcumin exerts anti-CCA activity in part through targeting multiple oncogenic signaling pathways [13] including nuclear factor kappa B (NF-κB), activator protein-1 (AP-1), signal transducer and activator of transcription-3 (STAT3) and protein kinase B (Akt) [2]. These findings support the use of curcumin as an alternative treatment, especially for CCA. However, establishing these facts requires very extensive and laborious experimental work using *in vitro* and *in vivo* models. Therefore, instead of that, discovering of the critical proteins and/or signaling cascades that are potential for CCA development and those which their expression could be modulated by curcumin treatment might be easier and useful because we can apply “the drug or targeted therapy” to target or interrupt those molecules/signaling pathways. Proteomics is a powerful approach to study changes in the proteome in response to disease development and to therapeutic intervention. This approach has been widely used to discover biomarkers for, and therapeutic targets in, a number of diseases including cancers [14].

Herein, we applied proteomics to identify potential protein targets for development of novel therapeutics for CCA. As part of a larger study [15], we utilized dietary *N*-nitrosodimethylamine (NDMA) in combination with *O*. *viverrini* infection to establish the CCA in Syrian hamsters (*Mesocricetus auratus*). Some of these hamsters were fed with a curcumin-supplemented diet. The liver proteome was evaluated using isobaric tags for relative and absolute quantitation (iTRAQ). Following tandem mass spectrometry, 5 proteins were further validated by western blotting and immunohistochemistry. This study identified major protein modulation by curcumin, and points the way towards potential therapeutic targets for CCA.

## Materials and methods

### Reagents

Curcumin (97% purity) was supplied by Merck-Schuchardt (Hohenbrunn, Germany). Rabbit polyclonal antibodies against S100A6 (sc50409) and GAPDH (sc25778) were obtained from Santa Cruz Biotechnology (Santa Cruz, CA, USA). Rabbit polyclonal anti-plastin-2 (ab83496), 14-3-3 zeta/delta (ab51129), vimentin (ab137321) and rabbit monoclonal anti-lumican (ab168348) were purchased from Abcam (Cambridge, MA, USA). For use as a secondary antibody, goat anti-rabbit IgG conjugated with horseradish peroxidase (HRP) was obtained from ZYMED (Thermo Fisher Scientific Inc., Walthem, MA).

### Parasites

Naturally-infected dead cyprinoid fish, important 2^nd^ intermediate hosts of *O*. *viverrini*, were bought from local markets in Khon Kaen Province, Northeastern Thailand. Metacercariae, the infective stage of *O*. *viverrini*, were isolated from the fish using 0.25% pepsin-HCl as described elsewhere [12]. Metacercariae were examined microscopically to ensure that they were alive (there was a movement of the worm inside the cyst). Fifty live metacercariae were used to infect male golden Syrian hamsters (*Mesocricetus auratus*) as described elsewhere [12].

### Experimental design

**Fig 1:**
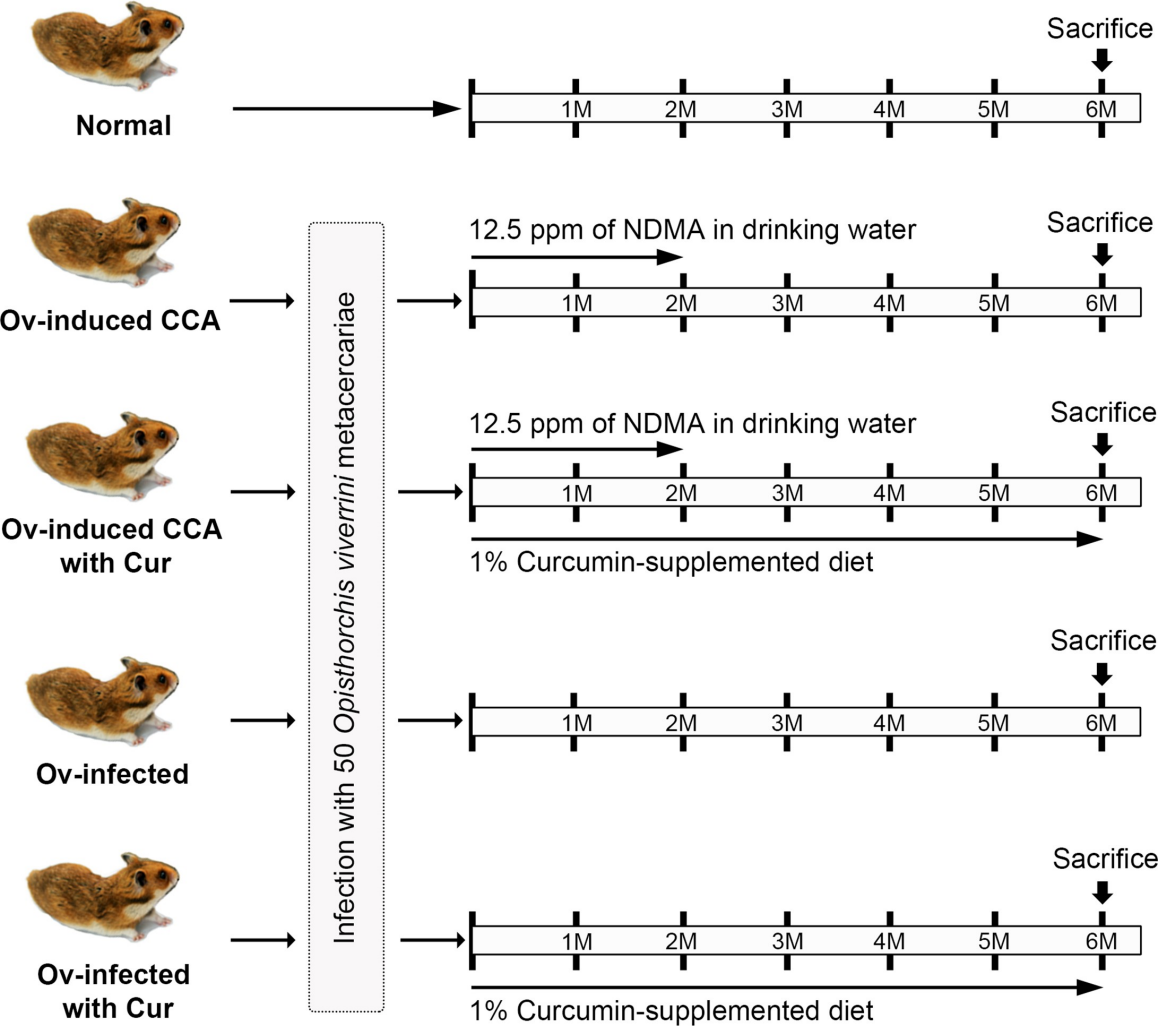
Experimental design. **A hamster model was used to study the effect of curcumin treatment on liver protein expression.** Five experimental groups of hamsters were used as shown in the diagram: 1) normal controls, 2) *Opisthorchis viverrini* (Ov)-induced CCA, 3) Ov-induced CCA with curcumin treatment, 4) Ov-infected and 5) Ov-infected with curcumin treatment. Administration of NDMA in combination with *O*. *viverrini* infection was used to induce CCA in hamsters. A diet containing 1% curcumin was given to hamsters in the “Ov-induced CCA with Cur” and “Ov-infected with Cur” groups for 6 months whereas the remaining groups were fed with a standard diet. After six months, hamsters were sacrificed, and the livers were subjected to iTRAQ and tandem mass spectrometry analyses.

This study was approved by the Animal Ethics Committee of Khon Kaen University, Thailand (AEKKU 22/2557). All surgery and necropsy were performed under ether anesthesia, and every effort was made to minimize pain and suffering to the animals. The experimental design is shown in Fig 1. Twenty male hamsters were housed under conventional conditions (12:12-h light:dark, temperature 25 ± 2°C), fed with a stock diet and given filtered bottled water *ad libitum*. Animals were randomly divided into five groups: normal controls; *O*. *viverrini-*infected and fed NDMA to induce CCA (CCA group); *O*. *viverrini*-infected and fed NDMA and 1% curcumin (CCA+Cur group); *O*. *viverrini*-infected only (OV group) and *O*. *viverrini*-infected with 1% curcumin supplementation (OV+Cur group). The protocol for CCA induction and administration of a 1% curcumin-supplemented diet has been described previously [12]. The health of hamsters was checked every 3 days. None of experimental hamsters exhibited severe illness or severe health problems during the study. All hamsters were anesthetized with diethyl ether inhalation and sacrificed by abdominal dissection and drawing of blood from the heart. After euthanasia, the liver from each hamster was collected and divided into 3 pieces for proteomics, western blot analysis, and immunohistochemistry. The liver samples for proteomic and western blot analyses were stored at -80°C until use.

### Protein isolation and purification from hamster livers

Protein was isolated from hamster livers and purified as described previously [15]. Briefly, 100 mg of liver tissue from each hamster in each group was suspended in 600 μl of lysis buffer (7 M urea, 2 M thiourea, 4% (w/v) 3-((3-cholamidopropyl) dimethylammonio)-1-propanesulfonate) and 40 mM Tris-base) and homogenized with a homogenizer at 4°C for 5 min. The samples were sonicated, solubilized and centrifuged at 12,000×g for 20 min at 4°C. Proteins were acetone-precipitated and then centrifuged at 8,000×g at 4°C for 10 min. The pellet was resuspended in 0.5 M triethylammonium bicarbonate (Sigma-Aldrich, Australia) and 0.1% sodium dodecyl sulfate (SDS). Protein concentration was measured by Bradford protein assay (Bio-Rad, Gladesville, Australia) according to the manufacturer’s instruction. Liver proteins from 3 hamsters of each group were used and considered as biological replicates for liquid chromatography-tandem mass spectrometry (LC-MS/MS) analysis.

### Protein reduction, alkylation and iTRAQ labelling

A total of 100 μg of liver protein from three hamsters in each group was reduced, alkylated and labelled using the iTRAQ Reagent-8PLEX Multiplex Kit (AB SCIEX, Mt Waverley, Australia) as described previously [15]. In brief, protein samples were reduced with 10 mM dithiothreitol at 60°C for 1 h and alkylated in 50 mM iodoacetamide at 37°C for 30 min in the dark. After digestion by trypsin with overnight incubation at 37°C, samples were labelled with iTRAQ reagents for 2 h at room temperature. Labeled peptides from all groups (the “CCA”, “CCA+Cur”, “Ov-infected”, “Ov-infected + Cur” and two control groups) were combined together, resulting in 3 biological replicates and each containing 6 peptide samples. Finally, the mixtures were sequentially passed through HiTrap ion exchange columns (GE Healthcare, Little Chalfont, UK), and SepPak C18 cartridge (Waters, Milford, MA, USA) to remove unbound iTRAQ reagent and to desalt. The purified peptides were then lyophilized and stored at -80°C prior to further analysis.

### Peptide OFFGEL fractionation

Purified peptides were fractionated using a 3100 OFFGEL Fractionator (Agilent Technologies, Santa Clara, CA, USA) with a 24-well immobilized pH gradient (IPG) strip (24 cm long, 3–10 linear pH range; GE Healthcare). Lyophilized peptide samples were reconstituted to a total volume of 3.6 ml using OFFGEL peptide sample solution, and 150 μl of mixture was loaded into each well. Isoelectric focusing was run with a maximum current of 50 μA until reaching 50 kVh. Twenty-four fractions were recovered, lyophilized and stored at -80°C prior to LC-MS/MS analysis.

### Tandem mass spectrometry

Lyophilized OFFGEL fractions were resuspended in 15 μl of 5% (v/v) formic acid in H_2_O and analyzed by LC-MS/MS using a Shimadzu Prominence Nano HPLC (Shimadzu, Brisbane, Australia) coupled to a Triple TOF 5600 mass spectrometer (AB SCIEX) equipped with a nano-electrospray ion source. In brief, 2 μl of each fraction were injected into a 50 mm × 300 μm C18 trap column (Agilent Technologies) at 20 μl/min and then de-salted on a trap column using 0.1% formic acid at a flow rate of 20 μl/min for 5 min. The column was placed in-line with the analytical nano-HPLC column (5 μm C18; 150 mm x 75 μm; Vydac, Theale, UK) for LC-MS/MS analysis. Peptide elution and ion-spraying were performed as described elsewhere [15]. Full scan TOF-MS data were acquired in an Information-Dependent Acquisition (IDA) mode over the mass range 350–1800 m/z and product ions 100–1800 m/z and then processed using Analyst TF 1.5.1 software (AB SCIEX).

### Protein identification and database searching

Spectral searching was performed using ProteinPilot v4 (AB SCIEX) with the Paragon algorithm [16] against the UniProt golden hamster (*Cricetulus griseus*) proteome database (UP000001075) and finally grouped using ProteinPilot’s ProGroup algorithm. The Trans Proteomic Pipeline (TPP) [17] with PeptideProphet and ProteinProphet was used to validate the peptides and identify the proteins. The Mayu algorithm [18] was used to calculate false discovery rate (FDR). The iTRAQ reporter ion intensities identified by ProteinPilot and which possessed a probability greater than 0.95 (S1 Table) were used in the R package iQuantitator [19] to estimate the credible intervals for protein expression across multiple iTRAQ experiments. The proteins were considered as up- or down-regulated if the start and end of computed 95% credible intervals were >1 or <1, respectively. Only proteins quantified on the basis of two significant peptides were considered.

### Soft clustering analysis

Soft clustering analysis was performed using R package “MFuzz” version 2.40 [20]. A set of 311 proteins having substantial changes in their expression either in one or more experimental groups were chosen for soft clustering analysis using the fuzzy c-means algorithm with default parameters.

### Western blotting

Proteins were extracted from hamster liver tissues (120 mg) using RIPA buffer (50 mM Tris/HCl, 150 mM NaCl, 1% Nonidet P-40, 0.5% sodium deoxycholate and 0.1% SDS) and protein concentration was determined using the Bradford assay. Ten micrograms of liver protein were separated by SDS-PAGE and transferred to a polyvinylidene difluoride membrane (PVDF, Amersham, Piscataway, NJ, USA) for 2 h at 60 V. After blocking with 5% skim milk in phosphate-buffered saline containing 0.05% Tween-20 (PBST), membranes were incubated with appropriate primary antibodies overnight at 4°C and then incubated with the HRP-conjugated secondary antibody for 1 h at room temperature. Chemiluminescent reaction was developed using ECL western blotting detection reagent (GE Healthcare) and then captured using the ImageQuant LAS4000 mini imager (GE Healthcare). The ImageQuant TL software v2005 (1.1.0.1) (Non-linear Dynamics, Durham, NC, USA) was used for densitometry of each band. Glyceraldehyde 3-phosphate dehydrogenase (GAPDH) was used as a loading control.

### Immunohistochemistry (IHC) and grading

An immunohistochemical study was performed using the immunoperoxidase method as described previously [15]. In brief, the hamster liver tissue sections were deparaffinized and rehydrated and antigens were unmasked by autoclaving for 10 min at 110°C with sodium citrate buffer (10 mM sodium citrate, 0.05% Tween-20, pH 6.0). After endogenous peroxidase quenching and blocking of non-specific binding, tissue slides were incubated at 4°C overnight with primary antibodies. After washing with PBS, tissue slides were incubated with the appropriate secondary antibody and the immunoperoxidase reaction was then developed with 3,3’-diaminobenzidine (DAB; Sigma-Aldrich). Staining density and intensity were placed in 4x4 grades, as described previously [15].

### Prediction and analysis of curcumin-protein interaction

STITCH software (http://stitch.embl.de), based on the following criteria: species (*Homo sapiens*), confidence score (0.40), and active prediction methods (all and no more than 10), was used to elucidate the potential interactions between each candidate protein (S100A6, lumican, plastin-2, 14-3-3 zeta/delta and vimentin) and curcumin as described previously [21].

### Statistical analyses

Relative protein expression levels are presented as mean±S.D. The differences of protein expression levels of each group were determined using the ANOVA. The differences of IHC grading scores among experimental groups were assessed by Kruskal-Wallis test. Statistical analyses were performed using SPSS version 15 (SPSS, Inc, Chicago, IL, USA). A *p*-value less than 0.05 was considered as statistically significant.

## Results

### Protein dysregulation in the liver of CCA hamsters and its possible prevention by curcumin

**Fig 2:**
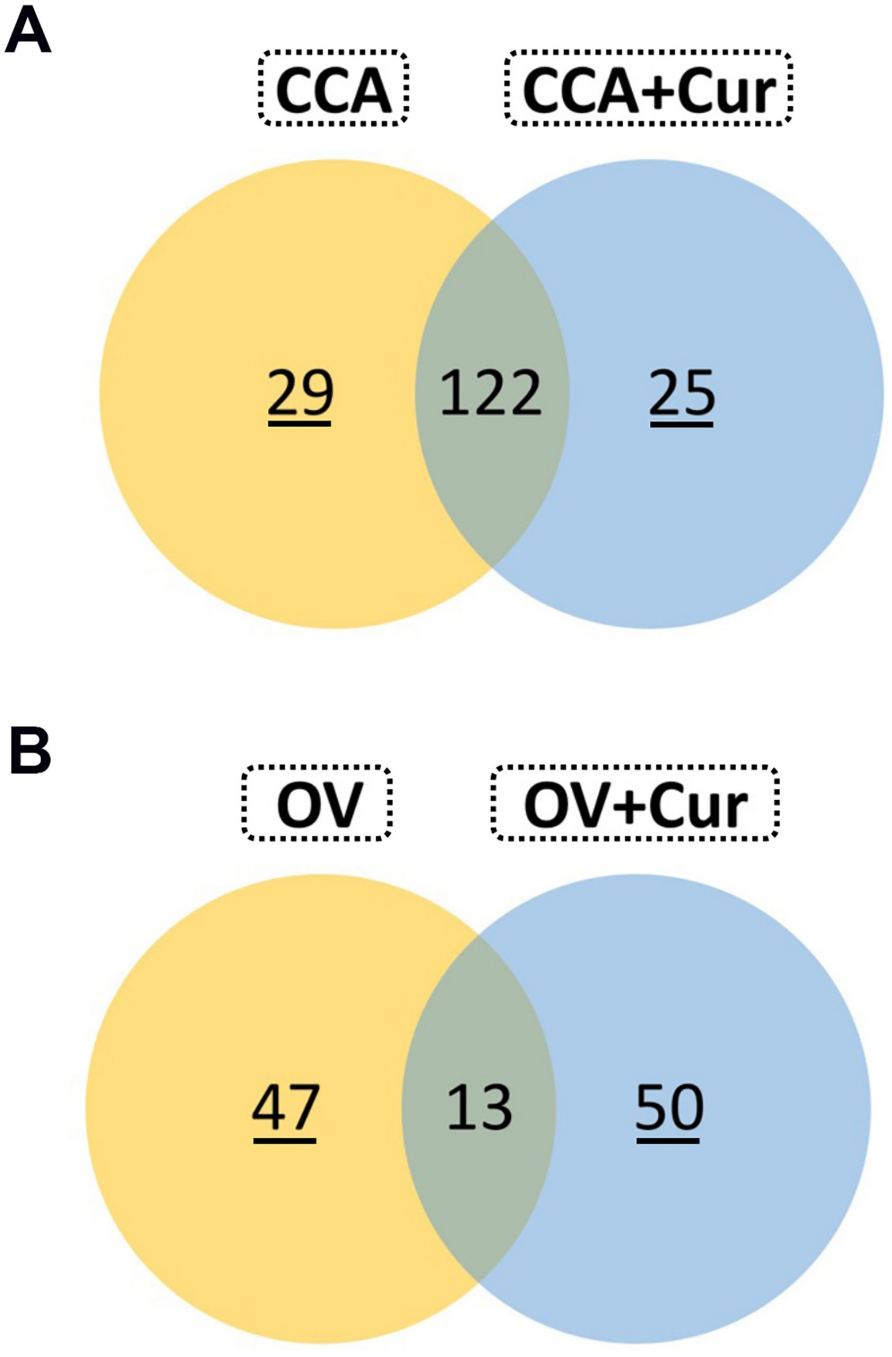
Differential protein expression in livers of hamsters in CCA vs. CCA+Cur groups and OV vs. OV+Cur groups relative to normal control livers. The expression of liver proteins in these hamsters was investigated by iTRAQ-mass spectrometry. The Venn diagram shows numbers of proteins dysregulated uniquely in (A) CCA vs. CCA+Cur groups and (B) OV vs. OV+Cur groups (relative to controls) and also those shared between the groups. The full list of proteins is shown in S3 Table.

Firstly, we sought to explore the overall expression of protein in the livers derived from hamsters of each experimental group. More than 500 proteins were identified in the livers of CCA hamsters. Two-hundred and forty-six proteins from hamsters in the CCA group and 262 proteins from hamsters in the CCA+Cur group significantly differed from those in the normal hamsters (S2 and S3A Tables). As shown in Fig 2A, 29 proteins in the CCA group (S3B Table) were expressed at significantly different levels than in normal and CCA+Cur livers and 25 proteins in the CCA+Cur group (S3C Table) significantly differed in expression levels from those in livers of normal and CCA groups. Although a further 122 proteins shared by the CCA and CCA+Cur groups differed significantly from the normal group in expression levels, their expression did not differ between the CCA and the CCA+Cur groups (S3D Table).

Interestingly, the expression levels of 99 proteins in the CCA group, which significantly deviated in expression levels from those in the normal controls, were also significantly modulated by curcumin treatment (S3E Table). Among them, 40 proteins, including 5 proteins previously identified in CCA hamsters [15] (S100A6, lumican, plastin-2, 14-3-3 zeta/delta and vimentin), were significantly upregulated in the CCA group, but significantly suppressed in the CCA+Cur group (Table 1). Some proteins, such as histone H3.1t, albumin, creatine kinase B-type, glutathione s-transferase theta-1, apolipoprotein A-I and peptidyl-prolyl cis-trans isomerase were upregulated in CCA hamsters, but their levels in the CCA+Cur group were comparable with those in normal hamsters (Table 1 and yellow labels in S3E Table). Conversely, 45 proteins were significantly downregulated in the CCA group, but were significantly upregulated in the CCA+Cur group (S3E Table). Four proteins were significantly upregulated in the CCA group and further upregulated in the CCA+Cur group (green labels in S3E Table) and another 10 proteins were under-expressed in the CCA group and even more decreased in the CCA+Cur group (orange labels in S3E Table).

**Table 1:** List of 40 upregulated proteins in CCA which were significantly suppressed by curcumin treatment.

No.	Accession	Description	Protein fold-change (Compared to normal)		CCA:CCA+Cur
			CCA	CCA+Cur	
1	G3IKI9	Serum amyloid A protein	7.00	3.03	0.47
2	G3HNJ3	Clusterin	6.16	2.80	0.48
3	G3I5L3	Annexin	4.88	2.77	0.62
4	G3HC31	Protein S100-A6	4.52	2.14	0.53
5	G3GTP7	Vitronectin	4.26	2.10	0.49
6	G3HJG6	Decorin	4.00	2.20	0.61
7	G3HSX8	Biglycan	3.75	1.64	0.48
8	G3I8F7	Keratin, type I cytoskeletal 19	3.73	1.67	0.49
9	G3HHR3	Vimentin	3.38	2.30	0.68
10	G3IG05	Annexin A2	3.32	2.26	0.71
11	G3I4Z7	Galectin-1	3.11	1.65	0.57
12	G3GVD0	Actin, cytoplasmic 1	2.89	1.50	0.53
13	G3HJG5	Lumican	2.89	1.59	0.58
14	G3I3Y6	Glutathione S-transferase P	2.85	1.81	0.67
15	G3HUU7	Protein S100-A10	2.73	1.51	0.62
16	G3II08	Keratin, type II cytoskeletal 7	2.70	1.66	0.64
17	G3H8N1	Plastin-2	2.62	1.93	0.78
18	G3HQC5	Prolargin	2.62	1.44	0.58
19	G3IDD4	Serpin H1	2.50	2.00	0.81
20	G3GZG6	Serotransferrin	2.45	1.62	0.67
21	G3HIX6	Tryptophanyl-tRNA synthetase, cytoplasmic	2.38	1.71	0.77
22	G3HPC9	Apolipoprotein E	2.38	1.14	0.49
23	G3H8Y5	Collagen alpha-1(VI) chain	2.38	1.92	0.83
24	G3HRQ4	Myosin light polypeptide 6	2.27	1.58	0.71
25	G3I1V3	Fibronectin	2.08	1.58	0.75
26	G3HKZ1	14-3-3 protein zeta/delta	1.98	1.44	0.77
27	G3I6I6	Tubulin alpha-1A chain	1.78	1.29	0.73
28	G3HG95	Lamin-A/C	1.74	1.39	0.80
29	G3HHM2	Histone H3.1t	1.72	1.18	0.74
30	G3I4H6	Fructose-bisphosphate aldolase	1.70	1.33	0.80
31	G3IDM2	Cofilin-1	1.70	1.26	0.75
32	G3HPV7	Histone H4	1.67	1.26	0.76
33	G3IAL6	Serum albumin	1.65	1.06	0.64
34	G3H377	Creatine kinase B-type	1.62	1.05	0.68
35	G3IMS5	Serum albumin	1.58	0.95	0.62
36	G3H576	Annexin A6	1.51	1.30	0.87
37	G3HY04	Glutathione S-transferase theta-1	1.40	1.02	0.75
38	G3IKC3	Glutathione S-transferase Mu 6	1.36	0.83	0.61
39	G3I7Q1	Apolipoprotein A-I	1.34	0.96	0.72
40	G3HIQ1	Peptidyl-prolyl cis-trans isomerase	1.19	1.05	0.89

### Effect of curcumin treatment on proteome changes in liver fluke-infected hamsters

*Opisthorchis viverrini*-associated CCA genesis is multistep and takes several years. Therefore, identification of proteomic changes during exposure to predisposing factors such as *O*. *viverrini* infection may improve knowledge of the mechanisms of tumorigenesis as well as identify possible chemopreventive targets for CCA. We therefore examined protein expression during infection with *O*. *viverrini* (OV group) compared with expression in the group infected with *O*, *viverrini* and given curcumin treatment (OV+Cur group) and in normal hamsters. Several proteins identified in both experimental groups were dysregulated relative to normal hamsters (S3F Table). As shown in Fig 2B, in comparison with normal hamsters, 47 proteins were dysregulated only in the OV group (S3G Table) and 50 proteins in the OV+Cur group (S3H Table). Thirteen proteins did not differ significantly in expression levels between the OV and OV+Cur groups, but did differ significantly between normal controls and these two treatment groups (S3I Table). Interestingly, the expression of 29 proteins, including lumican, vimentin and S100A6, were significantly upregulated in the OV group compared to normal controls, but upregulation was significantly suppressed by curcumin treatment (S3J Table). In contrast, 47 proteins were significantly downregulated in the OV group, but their expression was induced in the OV+Cur group (S3J Table). The expression of epoxide hydrolase 1 was significantly upregulated in the OV group compared to normal controls, and even more significantly in the curcumin treatment group, whereas the expression of Hsc70-interacting protein was significantly downregulated in the OV group compared to normal controls, and further reduced by curcumin treatment (S3J Table).

### Soft clustering of protein expression patterns across the experimental groups

**Fig 3:**
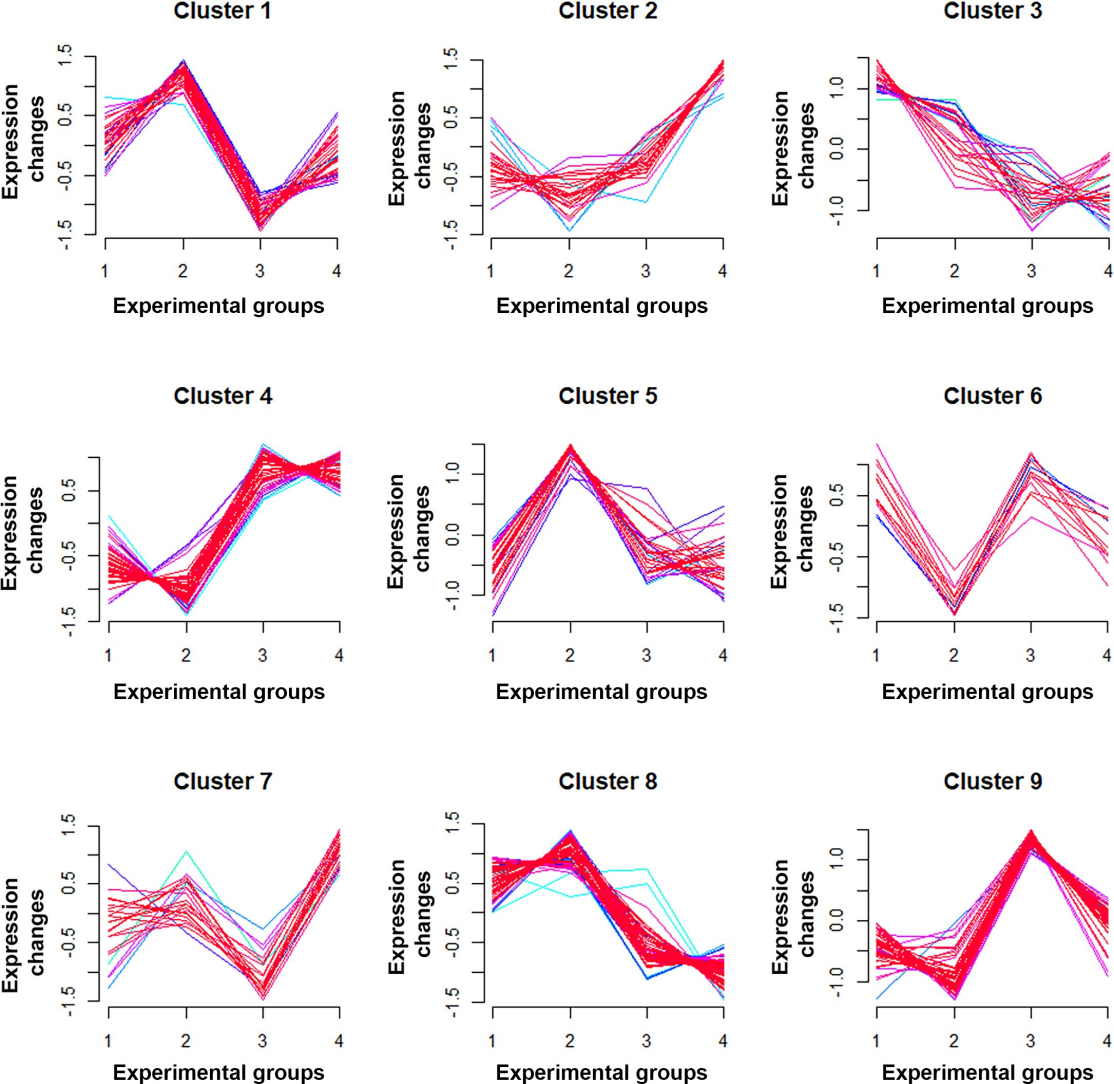
Cluster analysis of protein expression in all experimental groups. A significant dysregulation of proteins in at least one condition across all experimental groups was clustered by the R package Mfuzz into 9 different clusters. Clusters 6 and 9 represent the proteins that were upregulated in OV and CCA groups but were downregulated in the OV+Cur and in CCA+Cur groups, respectively. Clusters 1 and 7 indicate the proteins that were downregulated in the OV and CCA groups, but were induced in the OV+Cur and CCA+Cur groups. The experimental groups are numbered as follows; 1 = OV group, 2 = OV+Cur group, 3 = CCA group, and 4 = CCA+Cur group. The names of proteins in each cluster are shown in S4 Table.

Soft clustering analysis comparing the expression rations of 311 proteins derived from all experimental groups was performed. The proteins fell into 9 different clusters, according to differing expression profiles among the experimental groups (Fig 3). In total, 60 proteins fell into clusters 6 and 9 (Fig 3 and S4 Table). These clusters included proteins with significantly increased expression in the OV (experimental group 1 in S4 Table) and CCA groups (experimental group 3 in S4 Table) compared to the normal group, and which experienced suppression in the curcumin treatment groups (OV+Cur; experimental group 2, CCA+Cur; experimental group 4). Interestingly, some proteins in clusters 6 and 9, such as vimentin, lumican, S100A6, plastin-2, 14-3-3 zeta/delta, were previously identified as dysregulated proteins in the experimental CCA hamster model [15]. Apart from clusters 6 and 9, the expression of most proteins in clusters 1 and 7 (53 proteins; Fig 3 and S4 Table) was downregulated in the CCA group (group 3) compared to those in the OV (group 1) and OV+Cur (group 2) groups, but their expression was restored by curcumin treatment (CCA+Cur; group 4). Furthermore, the expression of proteins in cluster 2 (25 proteins; Fig 3 and S4 Table) also reflected the possible anticancer activity of curcumin as their expression was increased in the CCA+Cur group (group 4), compared to the OV (group 1), OV+Cur (group 2) and CCA (group 3) groups. Therefore, we suggest that the proteins in clusters 1, 2, 6, 7 and 9 might be good candidates for prevention as well as treatment of CCA.

### Curcumin treatment suppresses the dysregulated expression of proteins in liver of CCA hamsters

**Fig 4:**
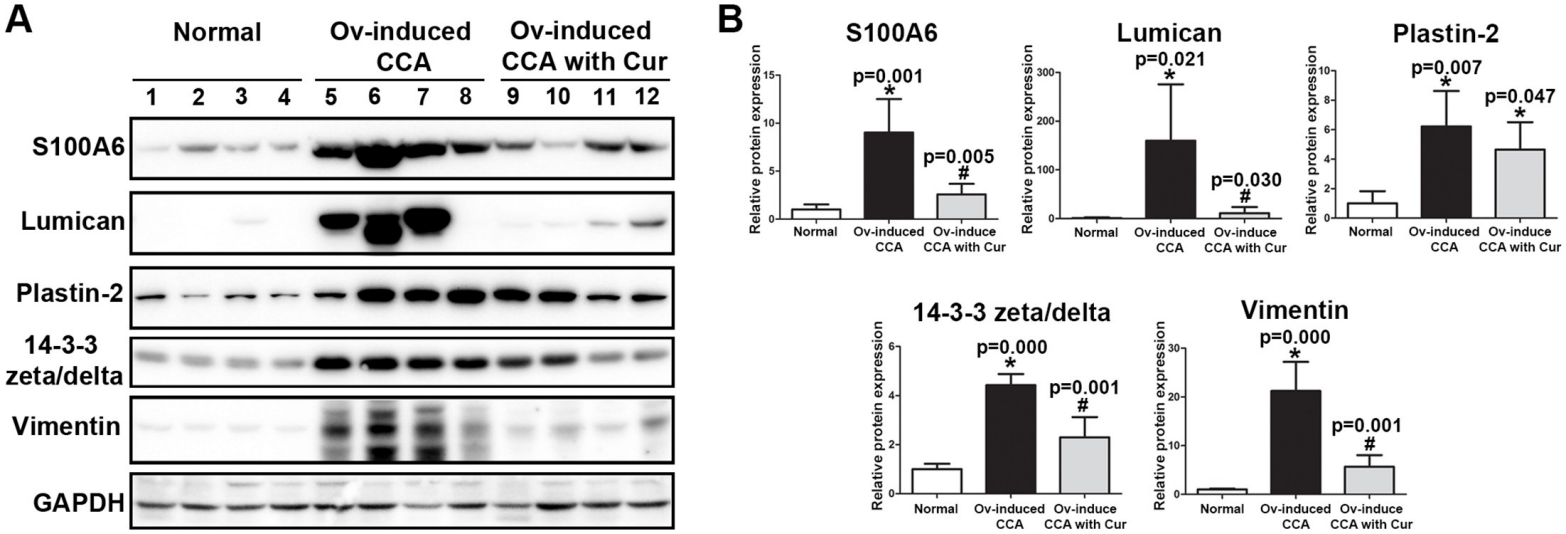
Western blotting validation of five candidate proteins (S100A6, lumican, plastin-2, 14-3-3 zeta/delta and vimentin) from iTRAQ protein expression data in hamster livers. (A) Western blot analysis detecting expression of candidate proteins in normal controls (lanes 1–4); the Ov-induced CCA group (lanes 5–8) and the Ov-induced CCA with curcumin treatment group (lanes 9–12). (B) The relative band intensities of the western blot analysis in the Ov-induced CCA group and the Ov-induced CCA with curcumin group were normalized by GAPDH and the results are shown as a bar graph. An asterisk (*) denotes a significant difference (*p* < 0.05) versus the normal group and a hash (#) denotes a significant difference versus the Ov-induced CCA group (*p* < 0.05).

Immunoblotting and immunohistochemistry (IHC) analyses were used to verify protein expression identified in the iTRAQ analysis. We previously established that the proteins S100A6, lumican, plastin-2, 14-3-3 zeta/delta and vimentin were significantly upregulated in CCA tissues derived from CCA hamsters and we hypothesized that all those proteins were involved in CCA genesis [15]. Furthermore, soft clustering analysis placed these proteins into clusters 6 and 9 because their levels were obviously increased in the CCA group compared to the normal and OV groups, but this overexpression was subsequently repressed by curcumin treatment (CCA+Cur group). We therefore verified the effects of curcumin treatment on the expression of those proteins in hamster CCA tissues. Consistent with the iTRAQ data, immunoblot analysis of hamster liver proteins confirmed a significant increase in the expression of all 5 proteins in the CCA group when compared to normal controls and 4 of the proteins (S100A6, lumican, 14-3-3 zeta/delta and vimentin) were significantly downregulated in the CCA+Cur group (Fig 4).

**Fig 5:**
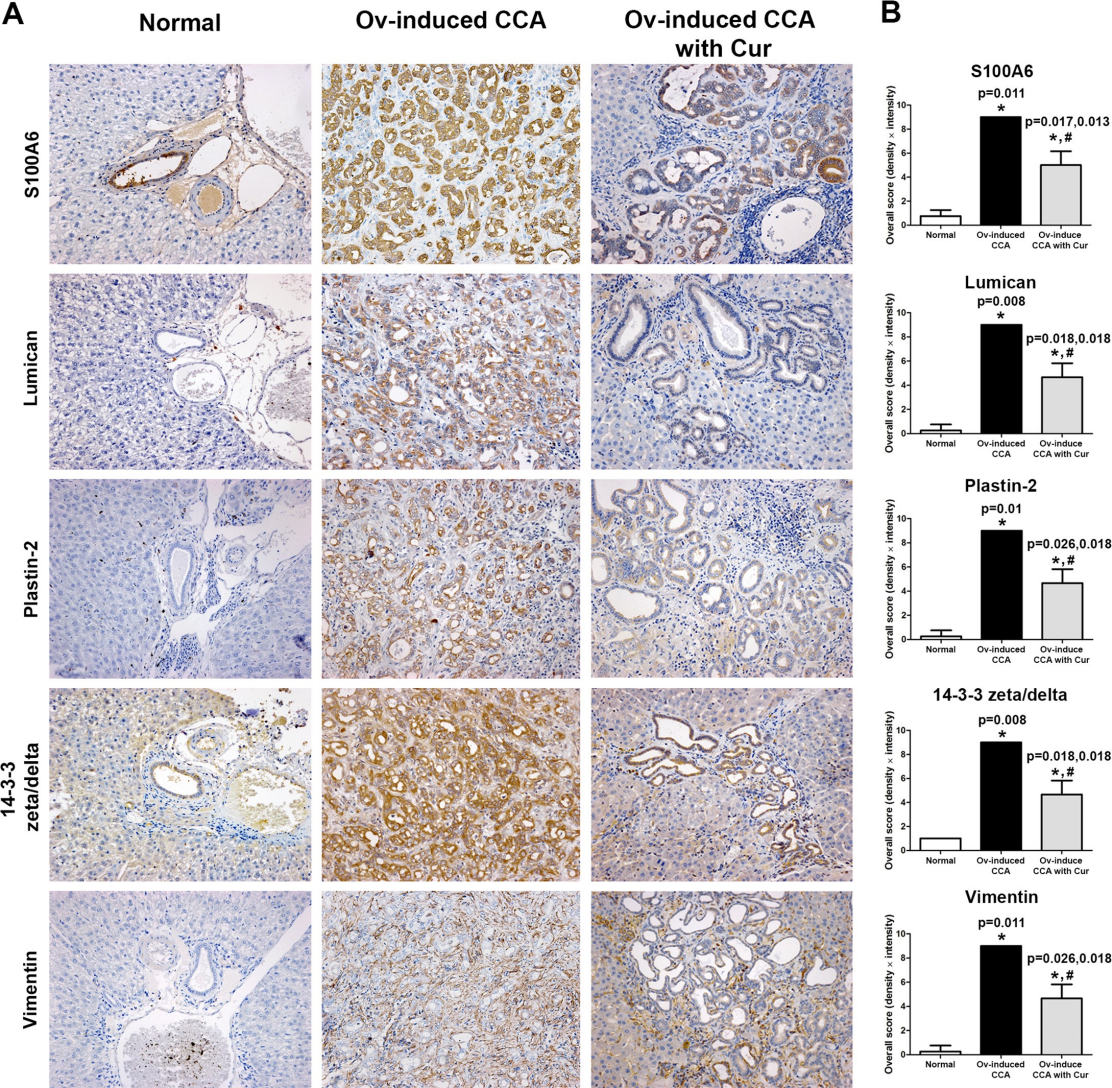
Immunohistochemical analysis of dysregulated proteins in hamster liver tissues. (A) The staining patterns of five proteins (S100A6, lumican, plastin-2, 14-3-3 zeta/delta and vimentin) derived from iTRAQ protein expression data in hamster livers. A representative section from each group is shown (4 cases/group; original magnification, ×200). (B) Overall expression scores were calculated from the intensity score multiplied by the density score. The overall grading scores are shown as a bar graph. An asterisk (*) denotes a significant difference (*p* < 0.05) versus normal controls whereas a hash (#) denotes a significant difference versus the Ov-induced CCA group.

Accordingly, IHC analysis revealed that S100A6, lumican, plastin-2 and 14-3-3 zeta/delta were expressed in the cytoplasm of tumor cells while vimentin was found mainly in the cytoplasm of fibroblasts in periductal fibrotic tissue and tumor stroma (Fig 5). Expression levels of these proteins were significantly higher in the CCA group compared to the normal group. Notably, although the levels of these proteins in the CCA+Cur group were significantly higher when compared to those in the control group, their expression levels were significantly lower when compared to the CCA group.

### Potential interaction of dysregulated proteins and interaction of curcumin with candidate proteins

**Fig 6:**
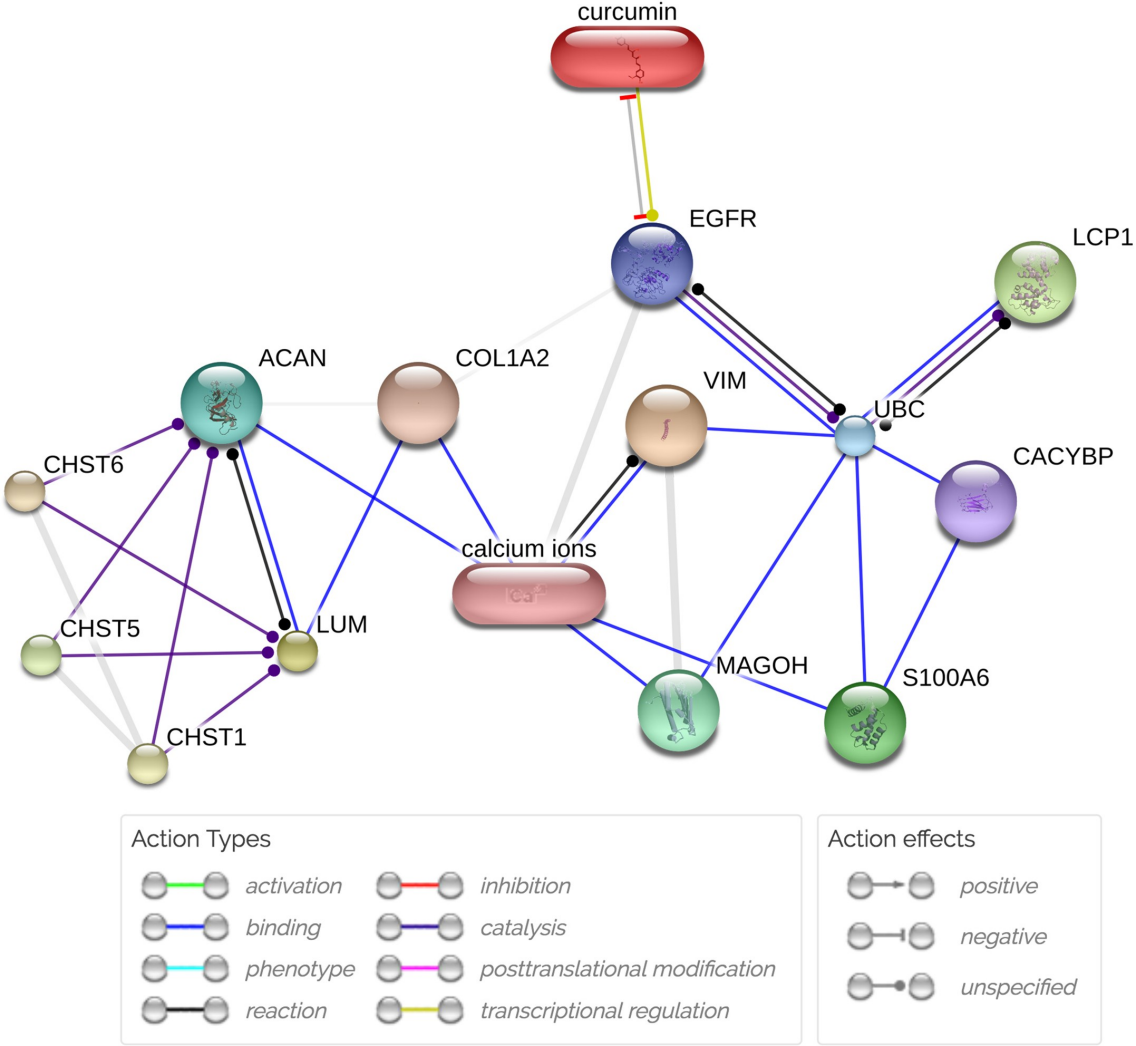
STITCH diagram of the protein interaction network between dysregulated proteins and curcumin. The network shows predicted interactions of candidate proteins (S100A6, lumican, plastin-2, and vimentin) and other dysregulated proteins with curcumin using STITCH analysis. Action types and action effects among dysregulated proteins and curcumin are illustrated. EGFR = epidermal growth factor receptor, UBC = ubiquitin C, LCP-1 (Plastin-2) = lymphocyte cytosolic protein 1, CACYBP = calcyclin binding protein, MAGOH = mago-nashi homolog, proliferation-associated protein, VIM = vimentin, COL1A2 = collagen, type I, alpha 2, LUM = lumican, ACAN = aggrecan, CHST6 = carbohydrate (N-acetylglucosamine 6-O) sulfotransferase 6, CHST5 = carbohydrate (N-acetylglucosamine 6-O) sulfotransferase 5, CHST1 = carbohydrate (keratan sulfate Gal-6) sulfotransferase 1.

The STITCH diagram (Fig 6) shows potential interactions between dysregulated proteins and curcumin for prevention and treatment of CCA. The dysregulated proteins likely had direct and/or indirect interactions with curcumin. For instance, curcumin showed a direct relationship with epidermal growth factor receptor (EGFR). EGFR is a protein in the HER/ErbB family and is involved in CCA progression mainly through induction of an epithelial to mesenchymal transition (EMT) and eventually invasion and metastasis [22]. In addition, curcumin indirectly interacted with S100A6, lumican, plastin-2, and vimentin through various pathways.

## Discussion

Although anti-inflammatory and anticancer properties of curcumin have been well studied in several chronic diseases [23, 24] and cancer models [25, 26], including in *O*. *viverrini* infection and CCA [12, 13, 27], the molecular mechanisms by which curcumin modulates protein expression and exerts its effects in CCA remain to be demonstrated. In this work, we used isobaric labelling to quantify protein expression in a hamster model of *O*. *viverrini*-associated CCA, with and without curcumin treatment. We primarily aimed at determining the mechanisms by which curcumin can interrupt the development of CCA and identifying potential therapeutic targets for treatment of CCA. *O*. *viverrini*-induced CCA arises after decades of chronic inflammation, which makes it similar to other inflammation-based cancers such as hepatocellular carcinoma (HCC), colon cancer and esophageal adenocarcinoma [28]. Accordingly, it is hoped that this work will also inform drug development efforts for other cancers induced by inflammation.

Several dysregulated proteins have been identified in livers of hamsters with *O*. *viverrini* infection and CCA. Among them, 5 proteins (S100A6, vimentin, lumican, 14-3-3 zeta/delta and plastin-2) were upregulated compared to normal hamsters, agreeing with a previous report using the same model [15]. Moreover, upregulation of these proteins was found in the OV group and their levels were persistently high until development of CCA, indicating the importance of these proteins in CCA genesis. The proteins S100A6 and 14-3-3 zeta/delta were also recently identified as abundant proteins in tumor interstitial fluid and cancerous tissue of CCA patients [29]. Interestingly, the expression of those 5 proteins was distinctly suppressed by curcumin treatment, especially in hamsters with CCA. S100A6, also called calcyclin, is reportedly involved in many aspects of cancers [30]. Overexpression of S100A6 is associated with poor prognosis of patients in many cancer types [31–33]. A recent study has shown that S100A6 activates the p38/MAPK pathway, leading to an increase of CCA cell proliferation, while silencing of S100A6 produces an opposite effect [34]. Vimentin [35] and lumican [36] have long been known as cancer-associated proteins and are involved in tumor growth and metastasis. Previous studies also showed that inhibition of vimentin’s activity or suppression of its expression induce cancer-cell apoptosis and inhibit the invasion, motility and migration of cancer cells [37–39]. Suppression of lumican also reduces tumor growth and metastasis in experimental animals [36]. The protein 14-3-3 zeta/delta is encoded by the YWHAZ gene [40] and has oncogenic potential in a number of cancers including CCA [41, 42]. Importantly, a high level of 14-3-3 zeta/delta expression is also associated with poor clinical outcomes of CCA patients [41, 42]. Plastin-2 belongs to the plastin protein family, which consists of 3 plastin isoforms. Of these, only plastin-2 or L-plastin is found in cancers [43]. Expression of plastin-2 induces proliferation, invasion and loss of E-cadherin expression [44], whereas suppression of plastin-2 diminishes progression and metastasis of cancer cells both *in vitro* and *in vivo* [45]. All this evidence is consistent with the hypothesis that curcumin suppresses CCA genesis, in part, by reducing expression of S100A6, vimentin, lumican, 14-3-3 zeta/delta and plastin-2.

The administration of curcumin during *O*. *viverrini* infection significantly affected the expression of other proteins involved in adhesion, fibrolysis and extracellular matrix degradation. All of these processes are well-known players during carcinogenesis. Many of the identified proteins are involved in wound healing and inflammatory process; vitronectin [46], myosin X [47], fibrinogen [48], delta-catenin [49], transgelin-2 [50], decorin [51] and clusterin [52]. These proteins were categorized in clusters 6 and 9 as they were overexpressed in both the Ov-infected and CCA groups and significantly repressed in the respective curcumin-treated groups. It has been proposed that cancer is able to ‘hijack’ the wound healing response to provide the stroma that is needed for their growth [53]. This is especially pertinent to proteins such as vitronectin, decorin and fibrinogen and suggests a potential anti-cancer ability of curcumin through suppression of key processes during CCA development. Clusterin is protein closely associated with activation of oncogenic transcription factor NF-κB [54]. NF-κB is believed to be an important key player in *O*. *viverrini*-induced CCA genesis [8, 55]. Constitutive activation of NF-κB is a feature this type of cancer and is found both in human CCA tissues [56] and human-derived CCA cell lines [13]. Suppression of NF-κB activation by curcumin treatment was previously demonstrated using both *in vitro* [13] and *in vivo* [12] models of Ov-induced CCA. Besides, the expression of other NF-κB activators, such as S100A10 protein [57], also increased in livers from hamsters of the OV and CCA groups and its expression was suppressed by curcumin treatment. Thus, suppression of clusterin as well as S100A10 might be among the mechanisms of CCA suppression by curcumin treatment. Other proteins in clusters 6 and 9, including annexin isoforms, cofilin-1, galectin-1 and coactosin-like protein, are also known to play different roles in cancers [58–61]. Therefore, they could also be the potential targets for prevention and treatment of CCA.

A number of proteins appeared to be over-expressed only in the CCA+Cur group. Proteins included in cluster 2 were over-expressed in the CCA+Cur group but were either under-expressed or not affected at all in the other experimental groups, suggesting a specific effect of curcumin on tumor cells. Some of these proteins have been identified as tumor inhibitors in some contexts, including calumenin [62], alpha2-macroglobulin [63], myosin II [64], heterogeneous nuclear ribonucleoprotein G [65] and nucleophosmin [66]. Conversely, a number of proteins traditionally regarded as oncogene or tumor markers, were also over-expressed in the CCA+Cur group, including the LIM and SH3 domain 1 protein, 78 kDa glucose-regulated protein and ribosome-binding protein 1. The 78 kDa glucose-regulated protein is redox-sensitive [67] and its upregulation in the CCA+Cur group suggests that curcumin is influencing the response of tumor cells to the local redox environment. Cells, including tumor cells, require a delicate balance of intracellular ROS since excess or insufficient of ROS is detrimental to cell functions and signaling pathways [68]. One of the main regulators of ROS is superoxide dismutase (SOD), an enzyme that undergoes dysregulation and functional abnormality in several cancer types [69]. SOD was originally considered a tumor suppressor as many tumors exhibited under-expression of MnSOD. Increased SOD expression interrupts superoxide anion/hydrogen peroxide balance, changes of cellular redox state and eventually suppresses cell proliferation [70, 71]. In this work, SOD was overexpressed in the CCA+Cur group. Therefore, upregulation of SOD in CCA+Cur hamsters might be responsible for the anti-CCA activity of curcumin, agreeing with previous studies in breast cancer [71] and prostate cancer [72]. Apart from SOD, upregulation of other antioxidant enzymes such as glutathione s-transferases and peroxiredoxin-1 (S3A Table), was also observed in livers of the curcumin-treated CCA hamsters compared to untreated-CCA hamsters. Thus, induction of these antioxidants may also be involved in preventive effects of curcumin against Ov-induced CCA in hamsters.

Administration of sub-carcinogenic dose of NDMA along with *O*. *viverrini* infection is a well-known procedure to induce CCA development in hamster [73]. Nitric oxide production during chronic opisthorchiasis not only contribute to chronic inflammation but also endogenous generation of NDMA [74, 75]. Hepatic cytochrome P450 enzyme family (CYP450) is a well-known enzyme which play a role in detoxification of NDMA [76]. Previous studies reported that the CYP2A6, an isoform of CYP450, was upregulated in opisthorchiasis [77] and opisthorchiasis-associated CCA patients [78]. In this study, our proteomic data found that the expression of CYP2A6 in CCA and CCA+Cur groups was not significantly deviated from that in control group. However, the expression of other CYP450 isoforms, the CYP4A14 was significantly downregulated in CCA hamsters but restored to normal in CCA+Cur hamsters, suggesting a role for CYP4A14 in NDMA detoxification and anti-CCA mediated by curcumin treatment.

Although curcumin has shown diverse pharmacologic effects and is promising for cancer treatment, this compound has some important drawbacks, particularly its bioavailability [79, 80]. Approaches taken to solve this problem have been reported, such as incorporation of curcumin in liposomes or other nanocarriers and use of curcumin analogues [3, 81]. However, in this study, it is clear that curcumin had a satisfactory effect on prevention of CCA development and that it affects expression of several proteins. As discussed above, many of these are key proteins involved in both inflammation and cancer. Our data provide a basis for identification of candidate proteins for clinical chemoprevention and therapy of CCA. This supposition is supported by the evidence for various direct and indirect interactions of curcumin with the dysregulated proteins (Fig 6). Additionally, further study on the function and the involvement of the candidate proteins in Ov-associated CCA may support the potential use of these proteins as therapeutic targets for treatment of CCA.

## Supporting information

S1 Table Peptides used in iTRAQ quantitation from replicate experiments. (XLSX)

S2 Table Credible intervals for CCA group versus normal controls using iTRAQ quantification. (XLSX)

S3 Table Protein expression ratios in different experimental groups. (XLSX)

S4 Table List of protein expression in different clusters with membership values and standardized expression ratios. (XLSX)
